# Tietze Syndrome in a 41-Year-Old Patient Without Significant Comorbidities

**DOI:** 10.7759/cureus.59640

**Published:** 2024-05-04

**Authors:** Berley Alphonse, Michelande Elien, Walter Jean-Jacques, Ricarven Ovil

**Affiliations:** 1 Internal Medicine, University Notre Dame of Haiti, Port-au-Prince, HTI; 2 Public Health, Montreal University, Montreal, CAN; 3 Orthopaedics, Hôpital Sainte Therese, Hinche, HTI; 4 Medicine, University Notre Dame of Haiti, Port-au-Prince, HTI

**Keywords:** corticosteroid treatment, acute joint pain, young athlete, young adult male, tietze syndrome

## Abstract

Tietze syndrome is a relatively uncommon condition characterized by painful swelling localized in the costo-sternal, sternoclavicular, or costochondral joints. Despite its benign nature, Tietze syndrome can mimic more serious conditions, necessitating thorough evaluation and exclusion of differential diagnoses. Management typically involves non-steroidal anti-inflammatory drugs and corticosteroid therapy, with surgical intervention reserved for refractory cases.

This case of a 41-year-old athlete underscores the importance of considering Tietze syndrome in the differential diagnosis of acute chest pain, especially in younger individuals without significant comorbidities. By raising awareness and sharing our experience, we aim to contribute to improved recognition and management of this condition, ultimately enhancing patient care outcomes.

## Introduction

Tietze syndrome, first identified by Alexander Tietze in 1921, is a relatively uncommon condition characterized by painful swelling localized in the costo-sternal, sternoclavicular, or costochondral joints [1,2]. Its exact prevalence is not defined, often labeled as a rare disease. The etiology and mechanism remain largely unknown, although hypotheses suggest microtrauma from repetitive coughing and vomiting. Notably, recent research has shown Tietze syndrome in some patients after COVID-19 infection [3]. Differential diagnosis primarily includes costochondritis; despite its benign nature, Tietze syndrome can cause significant discomfort and mimic more serious conditions such as acute myocardial infarction. Classic diagnostic criteria includes inflammation signs, swelling, and unilateral involvement of the first and second costochondral joints, alongside the onset of intense pain exacerbated by movement. While most patients with Tietze syndrome do not experience complications, recurrent cases may necessitate advanced treatment options such as lidocaine injections, intercostal nerve blocks, or surgery in refractory instances [4]. Here, we have presented the case of a 41-year-old patient diagnosed with Tietze syndrome; through this case, we aim to heighten awareness among healthcare professionals about this condition and its appropriate management.

## Case presentation

**Figure 1:**
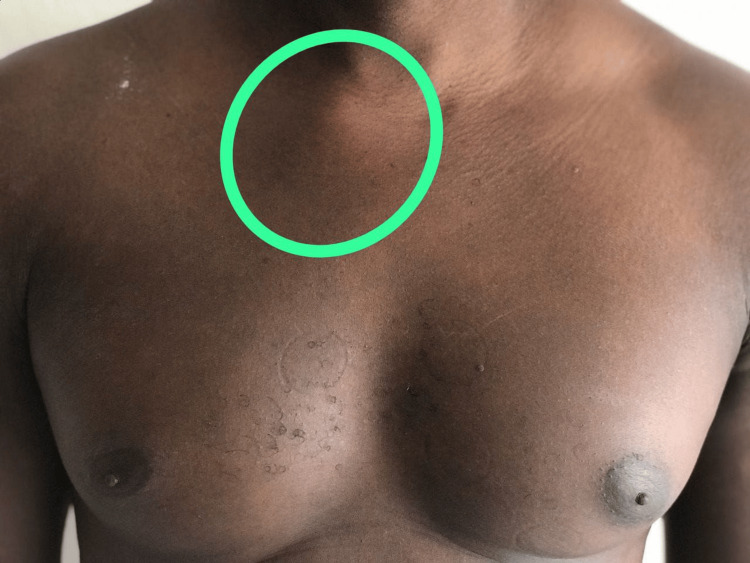
Swelling in the right sternoclavicular junction

**Figure 2:**
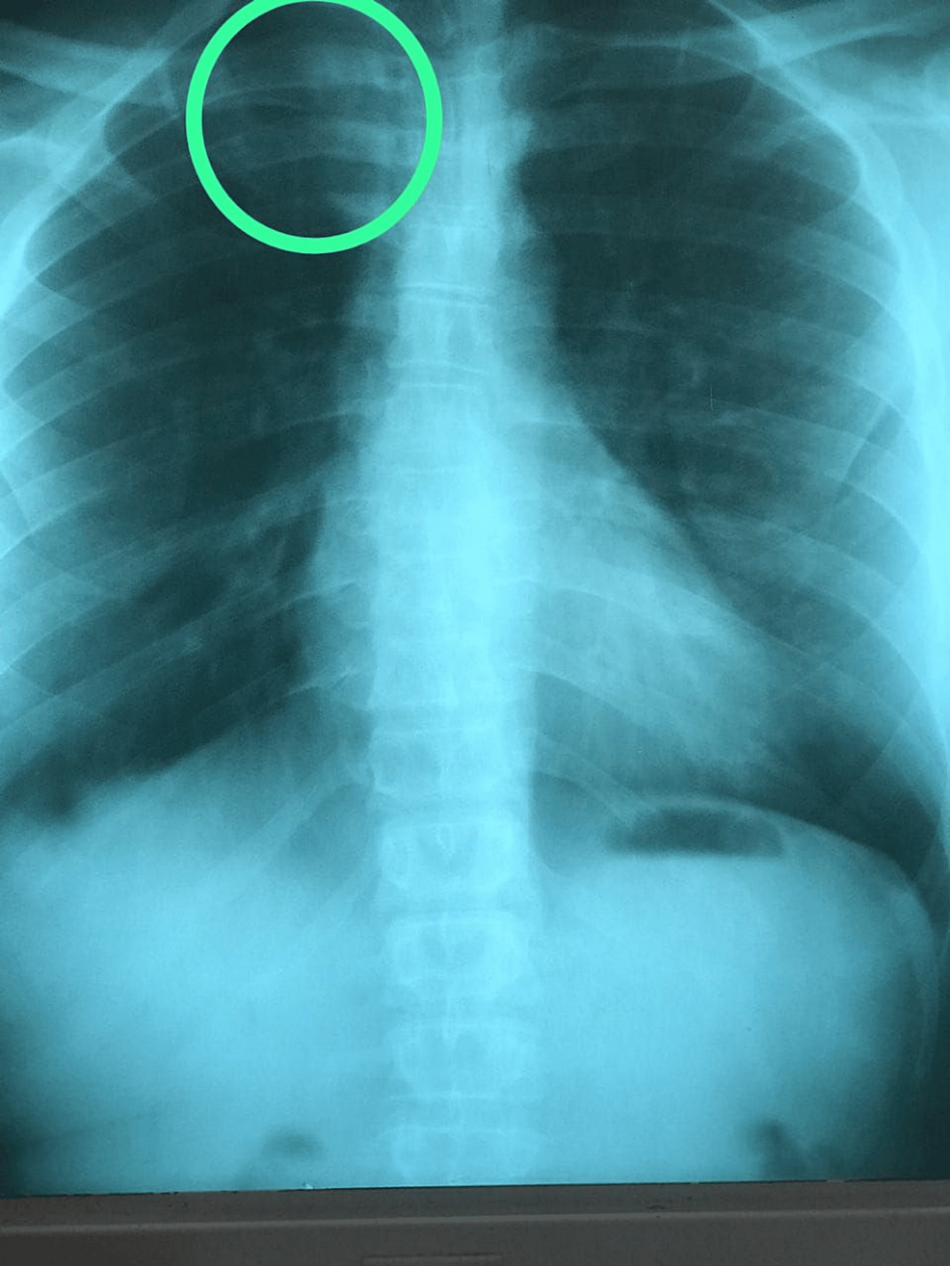
Inflammatory reaction at the right sternoclavicular junction

**Figure 3:**
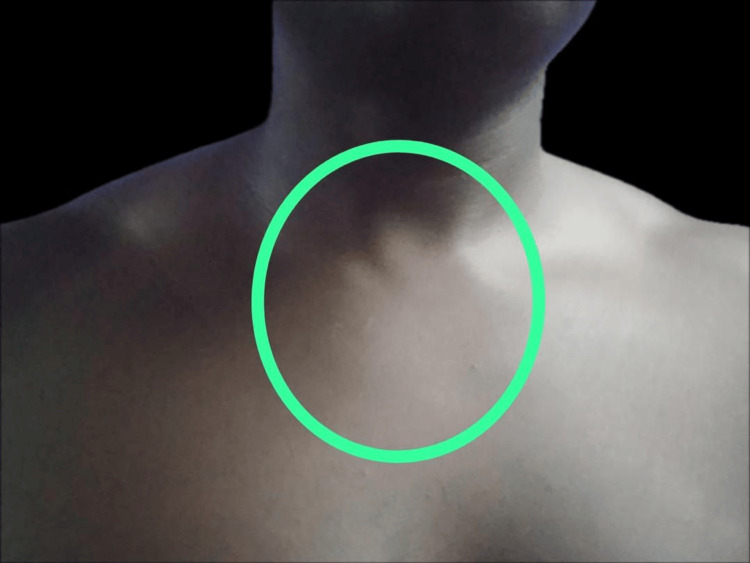
Complete resolution of the mass

A 41-year-old athlete presented to the emergency room with chest pain localized at the right sternoclavicular junction. The pain had been persistent for more than a few weeks, accompanied by swelling and functional limitation in the right arm and shoulder. There was no history of trauma, and the patient denied any comorbidities such as diabetes or hypertension. A physical examination revealed erythema, heat, and tenderness at the right sternoclavicular junction (Figure 1), along with pain on palpation and limited range of motion in the right arm. During the primary assessment, the patient’s vital signs were within normal limits, with clear airways and normal lung sounds bilaterally. The remainder of the physical examination was unremarkable, with no evidence of respiratory distress, cardiac murmurs, or palpable lymphadenopathy. However, there was a firm, well-circumscribed mass observable and palpable in the right sternoclavicular junction, which was painful with palpation. Chest X-ray showed a slight deviation of the thoracic spine and an increase in the diameter of less than 1 mm at the end of the right sternoclavicular junction (Figure 2); the electrocardiogram was normal, ruling out more serious cardiac or pulmonary conditions. Laboratory investigations revealed a slight increase in the white blood cell count, of 11,000/mm^3^, and elevated inflammatory markers, including an elevated sedimentation rate (42 mm/hour) and a positive CRP (48 mg/dl) test. Based on the clinical presentation, the diagnosis of Tietze syndrome was established, after excluding other causes of acute chest pain. The patient was prescribed injectable non-steroidal anti-inflammatory drugs (NSAIDs), and short-term corticosteroid therapy was initiated. After three months of follow-up, the sedimentation rate and CRP level returned to normal; he had no other complaints, and there was no more swelling in the right sternoclavicular junction (Figure 3).

## Discussion

Tietze syndrome primarily affects individuals under the age of 40, with a higher prevalence among women according to some studies [5,6]. However, conflicting evidence exists regarding gender predominance and seasonal variations in symptom onset [7]. Some studies suggest that males and females are affected by this condition in approximately equal proportions [4]. Our patient's case aligns with the typical demographic profile, being in the age group commonly affected by Tietze syndrome. He presented with erythema, heat, tenderness, and swelling of the right sternoclavicular junction, along with pain on palpation and limited range of motion in the right arm during active shoulder protraction. Notably, in myocardial infarction, swelling and inflammation of the costochondral joint are absent; chest discomfort and dyspnea may or may not be present. The pain does not worsen with movement; typically, it radiates to the face and left arm. The use of NSAIDs reduces the pain and nitroglycerine is avoided. On the other hand, in costochondritis, signs of inflammation are present (ESR, CRP); the pain is unilateral and occurs at rest [4].

The exact etiology and mechanism of Tietze syndrome remain poorly defined, complicating its diagnosis and management [4]. While hypotheses about microtrauma due to repetitive cough and vomiting exist, recent studies have highlighted the development of Tietze syndrome in some patients with COVID-19. The pathophysiology is believed to involve inflammation of the affected joints, leading to edema and pain. Management typically consists of NSAIDs and corticosteroid infiltrations [8,9], aiming to alleviate symptoms and reduce inflammation. Some studies have proposed treatment protocols, such as a three-week course of oral prednisolone or a regimen involving prednisolone and NSAIDs for varying durations [10-12]. In refractory cases, lidocaine injections, intercostal nerve blocks, and surgical intervention may be considered [13].

In our case, we followed a two-week treatment protocol. In the first week, the patient received 10 mg of oral prednisone daily and a 75 mg diclofenac intramuscular injection twice daily, followed by a second week of 5 mg oral prednisone daily and 75 mg diclofenac orally twice a day, resulting in a favorable outcome. The patient responded well to conservative management with NSAIDs and oral corticosteroids. It is noteworthy that our approach deviated slightly from the existing literature, utilizing a shorter duration of NSAIDs and prednisone treatment.

## Conclusions

Tietze syndrome is a rare but important differential diagnosis to consider in patients presenting with acute chest pain, especially in the absence of cardiac or respiratory symptoms. Our case underscores the significance of awareness among healthcare professionals regarding this condition, facilitating timely diagnosis; it also contributes to the existing literature, highlighting the need for a classification system for Tietze syndrome based on disease severity, especially considering the variety of treatment protocols available. By sharing our experience, we hope to contribute to the existing knowledge surrounding this disease and urge medical professionals to consider this illness when making a differential diagnosis for chest pain, improving patient care outcomes.
